# Autologous stem cell rescue recipient with neutrophil tissue delivery detected prior to blood engraftment: A case report

**DOI:** 10.1002/jha2.65

**Published:** 2020-08-07

**Authors:** Bushra Tbakhi, Fateeha Furqan, Glynis Scott, Jane L. Liesveld, Omar S. Aljitawi

**Affiliations:** ^1^ Department of Hematology and Oncology University of Rochester Rochester New York; ^2^ Department of Internal Medicine Rochester General Hospital Rochester New York; ^3^ Department of Dermatology and Pathology University of Rochester Rochester New York

**Keywords:** ANC, autologous, engraftment, neutrophil, skin

## Abstract

Neutrophil recovery after autologous hematopoietic cell transplantation (ASCT) is affirmed with achievement of an absolute neutrophil count (ANC) of ≥500/µL. There is growing evidence that neutrophils may be observed despite undetectable peripheral ANC counts following autologous hematopoietic cell transplant and are preferentially delivered to sites of inflammation. We report an interesting case that confirms neutrophil tissue delivery to the skin two days prior to evidence of blood engraftment after an ASCT.

## 1 INTRODUCTION

Myeloid engraftment is defined as an absolute neutrophil count (ANC) of ≥500/µL and is often achieved within 14 days of transplant [1, 2]. Potential methods of earlier identification of marrow recovery have been explored, although their clinical use is not yet widespread. We report a compelling case that demonstrates neutrophil tissue delivery, confirmed by skin biopsy, two days prior to evidence of blood engraftment in a patient who received an autologous hematopoietic stem cell transplantation (ASCT).

## 2 CASE REPORT

**FIGURE 1:**
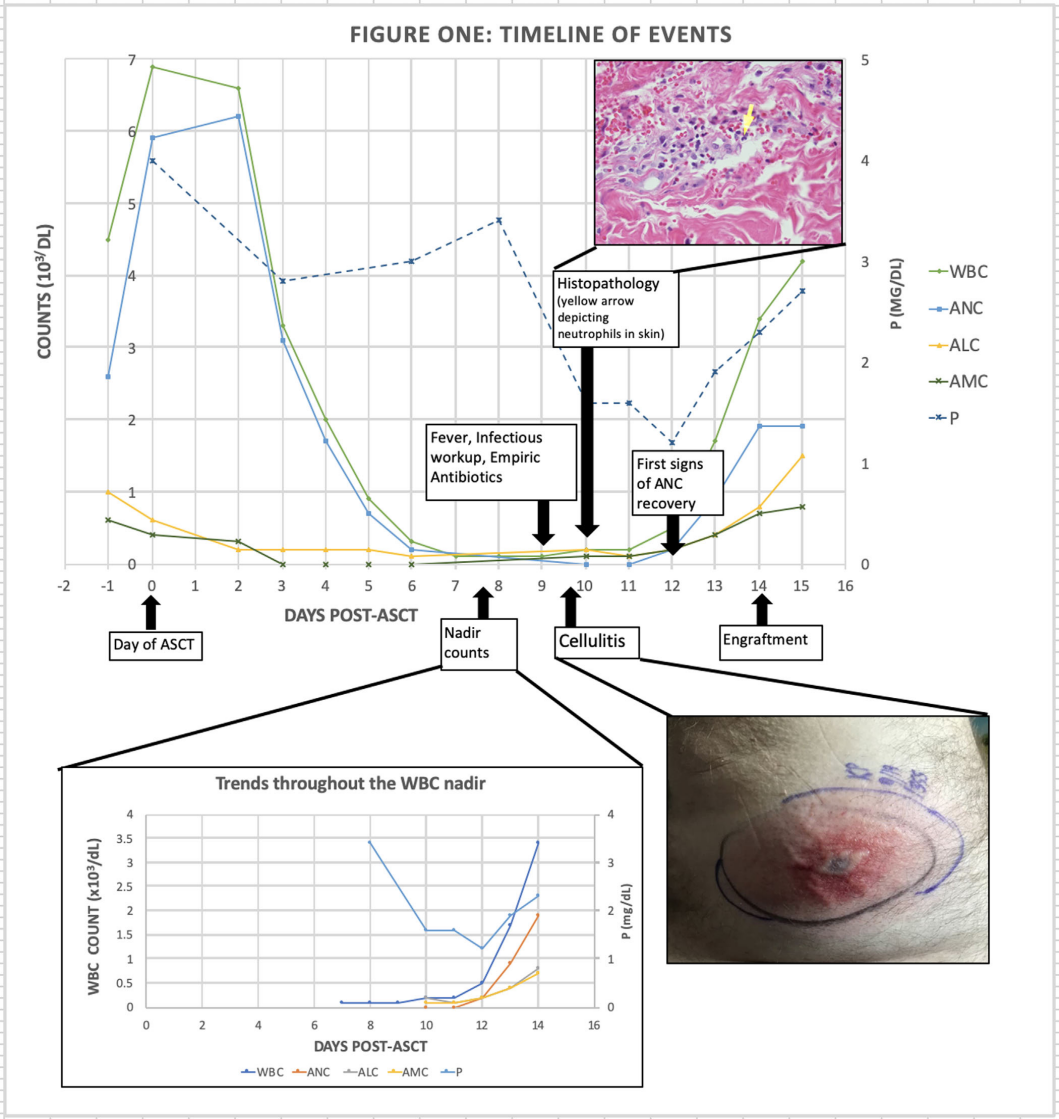
Timeline of events including trends in blood counts and phosphorus levels, clinical image depicting outlined area of cellulitis, and histopathology. WBC, white blood cell; ANC, absolute neutrophil count; ALC, absolute lymphocyte count; AMC, absolute monocyte count; P, phosphorous; ASCT, autologous stem cell transplant.

A 54‐year‐old man diagnosed with multiple myeloma received induction therapy with dexamethasone, bortezomib and cyclophosphamide, which was later changed to lenalidomide, bortezomib, and dexamethasone. After completing four cycles of chemotherapy with minimal side effects, he was referred for treatment with high‐dose chemotherapy followed by autologous stem cell rescue. He completed peripheral blood stem cell mobilization and harvest and received myeloablative conditioning with melphalan 200 mg/m^2^ on day −1. He was also enrolled into the hyperbaric oxygen (HBO) clinical trial (registered with Clinicaltrial.gov, number NCT03398200), which aims to investigate the effect of using HBO on the duration to neutrophil recovery after transplant and was randomized into the HBO cohort group. The patient underwent HBO therapy 6 h prior to his ASCT on Day 0, and then received the complete stem cell dose of 2.45 × 10^6^ CD34^+^ cells without any immediate complications. However, 8 days after transplant, he developed fevers and rigors. Blood work revealed leukopenia with zero ANC. Infectious work up for his febrile neutropenia including blood cultures was obtained and he was started on empiric intravenous cefepime and metronidazole.  Electrolytes were normal including a phosphorus of 3.4 mg/dL. On day 10 post auto‐HSCT, the patient developed an erythematous, tender swelling with induration on the left flank, without any history of preceding trauma (see Figure 1). Blood work still indicated an ANC of zero, absolute monocyte count of 100/µL and a phosphorus level of 1.6 mg/dL. Infectious disease service was consulted, and empiric vancomycin was added. However, the swelling continued to increase and developed discoloration prompting a dermatology consultation and a punch biopsy (Day +10). Both the gram stain and culture remained negative for bacterial growth, but the biopsy results revealed necrosis of the subcutis and mixed sparse dermal infiltrate of lymphocytes and neutrophils and some eosinophils as well as subepidermal edema.

On day 12 post ASCT, the patient achieved hematologic recovery with an ANC of 200/µL, and the phosphorus levels reached a nadir of 1.2 mg/dL. His fevers and cellulitis improved. He was discharged on the 14th day posttransplant with oral moxifloxacin as well as topical mupirocin. The patient was seen as outpatient with improvement in the cellulitis and no further febrile episodes.

## 3 DISCUSSION

Our case illustrates that neutrophil tissue delivery was indeed present prior to myeloid engraftment as his ANC was zero on the day of the biopsy of the skin lesion that demonstrated neutrophils in the tissue. Detection of ANC in this way is clearly not a replicable method of predicting early engraftment but does support the existing notion that marrow recovery occurs prior to the rising of an ANC level.

This incident also demonstrates that neutrophils mobilized to the site of inflammation, similar to the early delivery of neutrophils to the oral cavity. The presence of salivary neutrophils is in response to mucositis, a side effect of chemotherapy, which constitutes a common site of injury in this particular patient population [3]. However, there is evidence to support the rise of oral neutrophils in addition to blood neutrophils occurs by merit of inflammatory states not necessarily involving the oral cavity [4]. Of note, this patient was involved in the HBO clinical trial and was randomized to the interventional group, which may have affected the time to neutrophil recovery [5].

A variety of predictors of marrow recovery indicate that engraftment likely occurs earlier than the conventional evidence of an ANC level ≥500/µL. Examples of these markers include detection of neutrophils in oral mucosa, early monocyte recovery, hypophosphatemia, and measuring the immature reticulocyte population [6, 7, 8, 9]. Their use has suggested that marrow recovery after HSCT likely occurs at least 2 days earlier than blood engraftment. Oral rinses have been shown to detect neutrophils at a range of 2–8 days earlier than neutrophils in the circulation [6, 10, 11]. While this is a noninvasive method, their collection may be limited by presence of oral mucositis and nausea [11]. Absolute monocyte count ≥100/µL has been suggested to antedate ANC by about 5 days [7, 12]. The use of blood cell parameters (including volume, conductivity, and light scatter) showed earlier engraftment by approximately 4 days in both autologous and allogenic transplant groups, with the only difference between them being related to the volume of stem cells infused [12]. Hypophosphatemia (a drop in levels by 20%) is an electrolyte that can also reflect marrow recovery due to cell replication, although this is nonspecific and can be influenced by iatrogenic supplementation [7, 9]. In this particular case, hypophosphatemia, specifically a drop of 47% from baseline occurred congruently with the evidence of tissue neutrophil delivery, monocyte recovery, and prior to ANC recovery. Immature reticulocyte fraction, a tool that reflects erythroid engraftment and therefore marrow recovery and may be the earliest indicator of marrow recovery, preceded ANC recovery in ASCT by 3–6 days [7, 8].

Neutrophil repopulation is an essential milestone in the posttransplant period as the nadir period signifies a vulnerability of the immune system to infection. Our case supports the developing notion that engraftment in ASCT indeed occurs earlier than our current understanding that is based on monitoring ANC. The implications of confirming earlier engraftment in clinical practice may lead to shorter hospital durations, more judicious use of antimicrobial therapy, and perhaps a shift in investigating patients with late onset febrile neutropenia in the posttransplant period. Novel tools of predicting marrow recovery may be combined with the existing method of ANC monitoring or may serve as a stand‐alone tool, however further randomized prospective clinical trials would be needed before a tangible change in clinical practice can occur. Furthermore, findings involving autologous stem cell rescue such as this case may not be generalizable to allogenic stem cell transplants.

## CONFLICT OF INTEREST

The authors declare that there is no conflict of interest.

## INFORMED CONSENT

Written informed consent was obtained from the patient for publication of this case report and accompanying images. A copy of the written consent is available on request.

## AUTHOR CONTRIBUTIONS

Bushra Tbakhi and Fateeha Furaqan designed the research, interpreted the data, and wrote the paper. Glynis Scott contributed to data acquisition and interpretation. Jane L. Liesveld and Omar S. AlJitawi revised the manuscript critically for important intellectual content.

## Data Availability

Data sharing is not applicable to this article as no new data were created or analyzed in this study.
